# Exploring the behavior of *Candida antarctica* lipase B in aqueous mixtures of an imidazolium ionic liquid and its surfactant analogue

**DOI:** 10.3389/fchem.2023.1289398

**Published:** 2024-01-10

**Authors:** Paola R. Campodónico, Cristian Calderón, Jackson J. Alcázar, Belén Olivares, Limberg Jaldin, Cristian Suárez-Rozas

**Affiliations:** ^1^ Centro de Química Médica, Instituto de Ciencias e Innovación en Medicina, Facultad de Medicina, Clínica Alemana Universidad del Desarrollo, Santiago, Chile; ^2^ Facultad de Química y Biología, Universidad de Santiago de Chile, USACH, Santiago, Chile

**Keywords:** ionic liquids, enzyme, surfactant, catalysis, superactivity

## Abstract

The performance of *Candida antarctica* lipase B (CALB) has been evaluated in 1-butyl-3-methylimidazolium tetrafluoroborate (BMIMBF_4_)/water mixtures in a wide range of molar fractions ( $\chi_{BMIMBF4}$ ) with and without 1-dodecyl-3-methylimidazolium tetrafluoroborate (C_12_-MIMBF_4_), a surfactant derived from BMIMBF_4_. The main aim of this work is to evaluate the influence of $\chi_{BMIMBF4}$ over micellar aggregates to assess the activity of enzymatic reactions. The investigated reaction corresponds to the hydrolysis of the substrate *p*-nitrophenyl laureate in each $\chi_{BMIMBF4}$ . The kinetic study for $\chi_{BMIMBF4}$ at around 0.2 proved to be a border point in enzymatic activity. At $\chi_{BMIMBF4}$ = 0.1, the lipase activity increases in the presence of C_12_-MIMBF_4_. However, at higher concentrations, BMIMBF_4_ has a negligible effect over the lipase activity. These results suggest specific interactions between water and BMIMBF_4_ molecules in relation to CALB. This research highlights the superactivity phenomenon driven by the reaction media and the micelle interface. In this interfacial interaction, BMIMBF_4_ acts directly on the changes induced on the enzyme upon its interaction with the micellar interface. This study opens a green perspective toward the biocatalysis field.

## 1 Introduction

Natural resources facilitate reactions under gentle conditions. Enzymes, derived from readily available sources, serve as biodegradable, non-hazardous, and non-toxic catalysts. Typically, enzymatic reactions occur under mild conditions, such as physiological pH, room temperature, and atmospheric pressure. Leveraging enzymes in processes proves to be environmentally appealing, cost-effective, and sustainable.

In addition, biocatalysis considers at least 10 of the 12 principles of green chemistry (GC) (Sheldon and Woodley, 2018). GC, also known as sustainable chemistry, is not a particular set of technologies, but rather an area of study that emphasizes on the design of chemical products and processes with the aim of strongly reducing or eliminating chemicals that may become hazardous when transferred to the environment as waste (Sheldon, 2000). Catalysis is involved in i) highly selective and short synthesis and ii) products of high purity from a process that is efficient in energy with less waste compared to non-GC processes (Sheldon, 2016). Therefore, catalysts play a meaningful role in GC: i) decreasing energy requirements; ii) increasing selectivity; iii) diminishing hazardous conditions; and iv) minimizing side products (Sheldon, 1997; Sheldon et al., 2007; Vekariya, 2017).

Lipases are a sub-class of enzymes within the esterase family whose natural function is to hydrolyze long chains of oils and fats (Schomburg et al., 1991; Fojan et al., 2000). Hydrolytic enzymes have found widespread application in organic synthesis as ecofriendly catalysts with versatile substrate specificities. They exhibit high stereoselectivity, operate under mild reaction conditions, are readily available commercially, and do not require cofactors (Dolman et al., 1997; Ventura et al., 2012). Among these enzymes, *Candida antarctica* lipase B (CALB) stands out as one of the most effective catalysts, recognized for its exceptional stability compared to other lipases. CALB, a monomeric protein composed of 317 amino acids, belongs to the α/β-hydrolase fold family. Its active site comprises serine, asparagine/glutamate, and histamine. Notably, CALB distinguishes itself from most lipases by lacking a lid covering the entrance to its active site. Demonstrating efficiency, CALB is a catalyst for hydrolysis in water and esterification in certain organic solvents (Wu et al., 2013; Rabbani et al., 2015).

Water is considered the greenest solvent based on its chemical nature and quantity. However, some enzymatic reactions that contain hydrophobic substrates cannot take place in aqueous media (Xu et al., 2017). On the other hand, the removal of water from catalytic processes that proceed in aqueous media is extremely expensive due to its high boiling point (Sheldon and Woodley, 2018), which creates the need for water replacement toward conventional organic solvents (COSs). Hence, COSs have been used in biocatalysis to increase enzyme stability, improve the solubility of hydrophobic reagents, and to prevent unwanted side reactions (Zaks and Klibanov, 1984). However, COSs are highly volatile due to their significant vapor pressure, flammability, and toxicity. Moreover, the inhibitory activity rates related to the enzyme are much lower in COSs than in water (Zaks and Klibanov, 1984; Carrea and Riva, 2008; Sheldon, 2016; Xu et al., 2017). Khmelnitsky et al. (1994) reported that the enzymatic activity in COSs can be increased by lyophilization with large amounts of salt (KCl). An alternative to the COS are ionic liquids (ILs). Further studies of enzymatic catalysis in room-temperature ionic liquids (RTILs) have shown increases in their rate coefficients compared with COSs (Itoh, 2017). RTILs are molten salts composed entirely of cations and anions that melt below 100°C (Welton, 1999; Weingärtner, 2008) with remarkable physicochemical properties, i.e., being non-flammable, non-corrosive, and non-volatile and bulk physical constant, which can be tuned by combining different cations and anions (Freemantle, 1998; Chiappe et al., 2007). High combinatorial flexibility has converted these materials into “designer solvents” or “task-specific” solvents (Freemantle, 1998; Chiappe et al., 2007) whose properties can be specified to suit the requirements of a particular reaction (Reichardt and Welton, 2011). For these reasons, RTILs have gained importance in the biocatalysis field, being recognized as a very promising reaction medium. RTILs have shown that enzymes have the same catalytic behavior compared to water and COSs, improving enzyme selectivity, activity, and stability and preventing unwanted side reactions (Xanthakis et al., 2006; de Gonzalo et al., 2007; Sheldon, 2016). Previous studies have shown that RTILs with hydrophobic anions are less denaturing than COSs displaying high catalytic activities, while hydrophilic RTILs depend on the anion/cation moieties and alkyl chains, displaying harmful effects on enzyme activity/stability (Khmelnitsky et al., 1994; Sheldon et al., 2007; Van Rantwijk and Sheldon, 2007).

Since the 1990s, several types of enzymatic reactions in non-aqueous media have been studied, searching for alternative reaction media with an impact on GC (Gupta, 1992; Ballesteros et al., 1995; Cheong et al., 2022; Xue et al., 2022; Migowski et al., 2023). These have mainly considered proteases and lipases (Gupta, 1992; Ballesteros et al., 1995). The results based on the rate of the enzymatic reactions highlight the key role of hydrophobicity and polarity of the environment (Laszlo and Compton, 2001). Studies in solvent effects in enzymatic catalysis are a complex process as differences in enzyme hydration (Halling, 1994) and solvation of the enzyme and substrate must be considered. So, the key role of solvent effects focuses on the enzymatic activity for each solvent studied (Halling, 1994; Klibanov, 1997; Eckstein et al., 2002), and there is great scope within this field yet to be explored. Sheldon et al. (2002) published a second article on enzymes in RTILs and first on CALB in 1-butyl-3-methyl imidazolium hexafluorophosphate (BMIMPF_6_) and 1-butyl-3-methyl imidazolium tetrafluoroborate (BMIMBF_4_) comparing those RTILs with some COSs (Lau et al., 2000). Currently, a great number of publications show that RTILs based on hydrophobic anions, such as BF_4_^−^, PF_6_^−^, and bis(trifluoromethylsulfonyl)imide (NTF_2_^-^) are less denaturing than some COSs, and they are responsible for higher catalytic activities (Van Rantwijk and Sheldon, 2007; Abe et al., 2008). However, hydrophilic anions, such as nitrate, acetate, or lactate anions, have a deleterious effect on the enzyme activity/stability by the formation of a strong hydrogen bond (HB) or Coulombic interactions (Sheldon et al., 2002; Lau et al., 2004). Therefore, anion studies based on RTILs have suggested that employing less polar RTILs may maintain a protective water layer around the enzyme, thereby contributing to its stabilization (Micaêlo and Soares, 2008; Attri et al., 2011). Perhaps, this first shell of solvation might play a key role in enzyme activity through the HB established between the enzyme and anion(RTIL) (Sheldon et al., 2002). So, the anions should be able to accept the HB in order to maintain the structural conformation of the enzyme, discarding small and charged anions able to penetrate the protein matrix, reducing the flexibility or mobility of the enzyme active site (Anderson et al., 2002; Sheldon et al., 2002). On the other hand, increasing the alkyl chain in the cation leads to an increase in the hydrophobicity and van der Waals interactions responsible for the partial or total obstruction of the active site of the enzyme hindering the substrate–enzyme interaction and reducing the lipase activity (Fan et al., 2016).

Three approaches to working with non-conventional solvents in biocatalysis are i) pure solvent; ii) co-solvent in aqueous systems, and iii) biphasic systems (Kragl et al., 2002). In general, the solvent effect over the catalytic performance is described as i) stripping off the water layer around the enzyme interface; ii) penetrating the micro-aqueous phase to interact with the enzyme in order to change the conformation and/or active site; and iii) interacting directly with substrates and products or modifying their partitioning between hydrophilic and hydrophobic phases (Yang, 2009; Ventura et al., 2012). In summary, the influence of the reaction media over the enzymatic reaction is studied in terms of improving selectivity, activity, and stability. This influence depends on the catalyzed reaction and nature of the enzyme under study. Therefore, it is significant to elucidate under what circumstances and how the biocatalyst preserves its biological function and stability in these solvents. Currently, the research on relationships between solvents and enzyme functions is a large field to explore in order to identify suitable solvents that ensure enzyme stability/activity.

Despite the green features of the ILs, it is worth highlighting their potential damage to the environment (Pernak et al., 2001; Amde et al., 2015). Gonzalves et al. (2021) evidenced that the effect of these non-conventional solvents exerts an action over different organisms, suggesting that a critical role is centered over the cation based on their lipophilicity feature compared to the minor role of the anion (Gonzalves et al., 2021).

**SCHEME 1:**
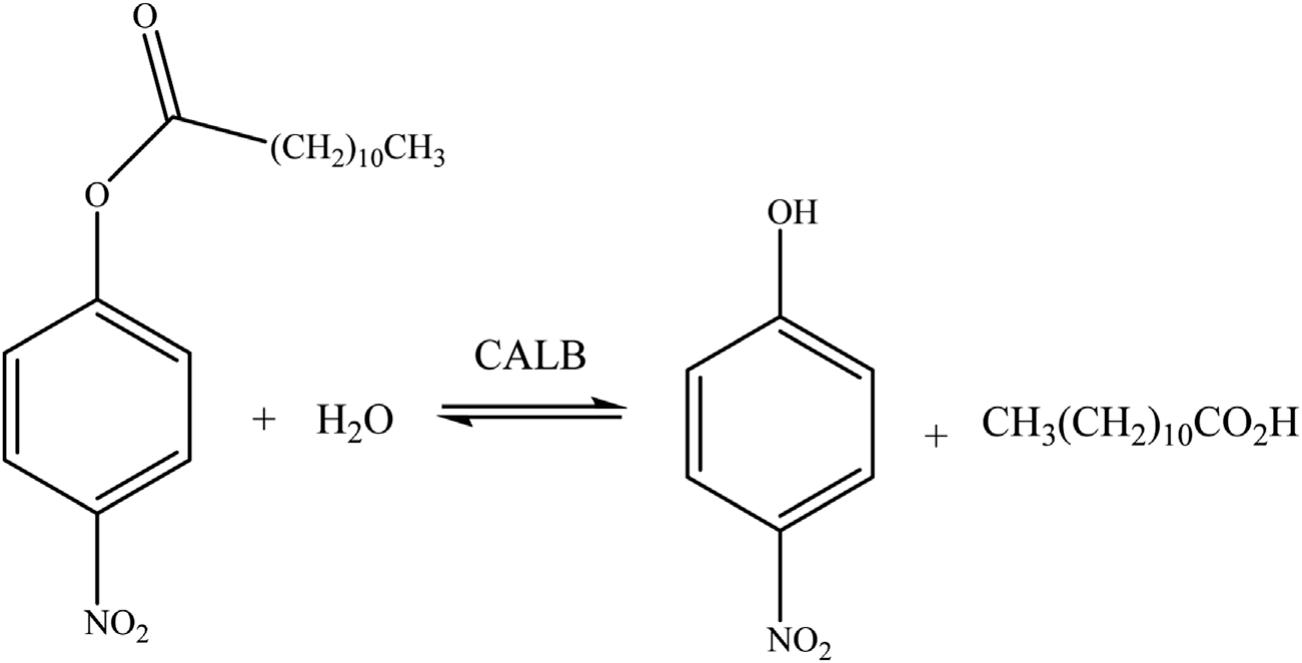
General picture of the hydrolysis reaction of the substrate *p*-nitrophenyl laureate (*p*-NPL) mediated by CALB.

More recently, ionic liquids have been used as a surfactant in order to improve the lipase activity. In fact, surfactants immersed in ILs are a promising reaction medium because the interactions established (surfactant–water–IL) are minimized due to i) the nature of the surfactant headgroup and counterions (Calderón et al., 2019) and ii) solvation effects (Wijaya et al., 2016; Vicent-Luna et al., 2017). Then, this work uses BMIMBF_4_ as a reference solvent and its long chain derivative, i.e., 1-dodecyl-3-methylimidazolium tetrafluoroborate (C_12_-MIMBF_4_), and our main aim is to evaluate the influence of molar fractions of BMIMBF_4_/water mixtures over micellar aggregates in order to assess the activity of the enzymatic reaction. The investigated reaction corresponds to the hydrolysis of the substrate *p*-nitrophenyl laureate (*p*-NPL) in each reaction medium (see Scheme 1 below). This work shows a comparative study of the activity of CALB in pure and solvent mixtures at different molar fractions of BMIMBF_4_/potassium phosphate buffer solution (considered water) and the same molar fractions with C_12_-MIMBF_4_, respectively.

## 2 Experimental section

### 2.1 Materials and methods

#### 2.1.1 Materials

BMIMBF_4_ and C_12_-MIMBF_4_ were purchased from Merck and IoLiTec, respectively. The specifications for BMIMBF_4_ were purity (HPLC) >98% and water (KF) < 0.1%. C_12_-MIMBF_4_ was not fully tested based on regulation (EC) 1272/2008. CALB, *p*-NPL (purity ≥98% by gas chromatography [GC]) 1, *p*-nitrophenol (*p*-NP, purity (DSC assay) > 99.5%), and dimethyl sulfoxide (DMSO, purity (GC) > 99.9%) were acquired from Sigma. The salts KH_2_PO_4_ and K_2_HPO_4_ were acquired from Merck (purities (alkalimetric assay) > 99.5%). All reagents were used as soon as delivered. Ultrapure water was used for the preparation of the aqueous solutions used (Merck Millipore Simplicity™ UV water purification system).

#### 2.1.2 Lipase activity assays

This study uses pure BMIMBF_4_ and phosphate buffer as the aqueous media, 50 mM and pH = 7.0, and BMIMBF_4_/buffer mixtures in a wide range of molar fractions (χ) with/without the presence of a surfactant derived from the same ionic liquid, C_12_-MIMBF_4_ (10 mM). Each mixture was prepared by weighting the proper amount of the IL and buffer in a screw-capped vial. To favor mixing, each mixture was shaken and sonicated for 1 min and then left to equilibrate overnight before use. In all cases, the mixtures appeared homogeneous after this treatment. In those mixtures with C_12_-BMIMBF_4_, it was added after to be shaken and sonicated.

The substrate solution (*p*-NPL) was prepared in DMSO at 58 mM, and it was directly injected (10 µL) in each reaction medium. Lipase activity was measured by UV–Vis spectrophotometry using an Agilent 8453 UV–Vis spectrometer. Aliquots from a stock solution (50 μL) of lipase were added to 2.5 mL of each reaction medium containing the *p*-NPL. The release of *p*-NP was recorded by following the increase in absorbance at 410 nm. The concentration of *p*-NP was determined from absorbance data using a calibration curve. The initial reaction rates were calculated during the first 150 s of the initial segment of the reaction profiles. The enzymatic solution was prepared by adding 10 mg by 1 mL of potassium phosphate buffer solution. From the plots of *p*-NP release vs. time obtained at different *p*-NLP concentrations while keeping the amount of CALB added to each kinetic experiment constant, the dependence of the initial reaction rates with *p*-NPL was established. The determination of the Michaelis–Menten kinetic parameters ( $k_{cat}$ and $K_{M}$ ) was performed according to the following equation:

$$v=\frac{k_{cat}\left[E_{0}\right]\left[S\right]}{K_{M}+\left[S\right]\;},$$ (1)

where v corresponds to the rates of CALB-catalyzed hydrolysis of *p*-NPL. $\left[E_{0}\right]$ is the enzyme concentration used in the hydrolysis experiments, and $\left[S\right]$ corresponds to the substrate concentration at which the associated reaction rate was determined (Calderón et al., 2019).

#### 2.1.3 Critical micelle concentration determination

Conductivity measurements were used to evaluate the critical micelle concentration (CMC) of C_12_-MIMBF_4_ using an Adwa AD3000 conductometer provided with a 4-pole conductivity probe. The conductivity of water (buffer phosphate), BMIMBF_4_, and BMIMBF_4_/water mixtures was briefly measured upon adding some stock solution (c.a. 50–200 μL) of C_12_-MIMBF_4_ (100 mM), prepared in the corresponding solvent mixture. CMC values were determined at the breaking point observed in the plots of conductivity, expressed in mS/cm vs. [C_12_-MIMBF_4_] in all the ranges of molar fraction with respect to BMIMBF_4_ (see Supplementary Figures S1, S2 in electronic Supplementary Material) (Evans, 1956).

## 3 Results and discussion

**FIGURE 1:**
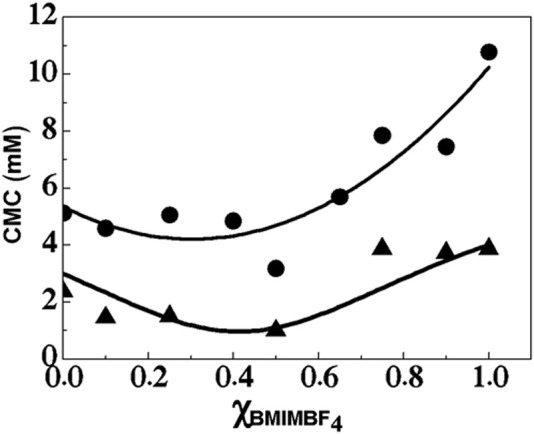
Variation in the critical micelle concentration (CMC) of C_12_-MIMBF_4_ with the molar fraction of BMIMBF_4_ in an aqueous solution, without *p*-NPL (•) and 0.58 mM *p*-NPL (▲).

In order to obtain useful kinetic information that can be compared with the data obtained in pure media and solvent mixtures, special emphasis was placed on the evaluation of the extent of the influence of the micellar aggregates at a fixed molar concentration of surfactant (10 mM). This concentration was used in order to ensure that the surfactant concentration is beyond the CMC, where the presence of micellar aggregates acquires relevance. Figure 1 shows the relationships between the variation in the CMC of C_12_-MIMBF_4_ and the molar fraction to respect to BMIMBF_4_ ( $\chi_{BMIMBF4}$ ). All the $\chi_{BMIMBF4}$ solutions were prepared in 50 mM buffer phosphate, pH = 7.0, and pure BMIMBF_4_. The CMC is in the range 4–10 mM of the surfactant without *p*-NPL (full circles in Figure 1). On the other hand, with *p*-NPL, the range is between 1 and 5 mM of the surfactant (full triangles in Figure 1).

Figure 1 shows two trends related to the variation in the CMC vs. IL content (in the presence and absence of the substrate), expressed as $\chi_{BMIMBF4}$ . For the first trend (full circles, without *p*-NPL), there is a decrease with the initial addition of the IL ( $\chi_{BMIMBF4}$ = 0.1); however, the CMC remains relatively constant until close to $\chi_{BMIMBF4}$ = 0.4. Past this point, the CMC values steadily increase, with a value close to 10 mM without *p*-NPL and a much lower value in the presence of *p*-NPL (close to 4 mM). Overall, the CMC is lower in the presence of *p*-NPL throughout the whole $\chi_{BMIMBF4}$ range. The same fact was reported by Ventura et al. (2012); Łuczak et al. (2015). This observation is particularly interesting, given the fact that the significant change in CMC is attributable to the cosurfactant behavior displayed by the substrate (up to a concentration of 0.58 mM), which is readily incorporated into the micellar moiety. On the other hand, this is also relevant as a consideration for the substrate concentrations used in the enzymatic assays (10^−5^–10^−4^ M) because this concentration ensures no significant changes are introduced in the micellar moiety due to the incorporation of the substrate.

**FIGURE 2:**
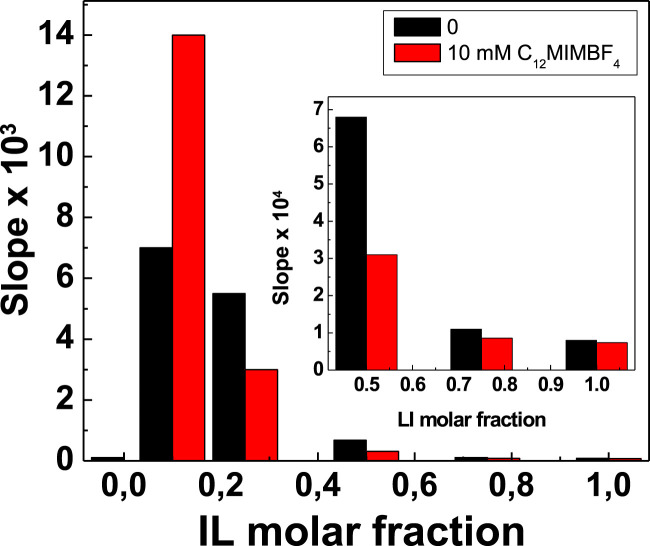
Reaction rate for the lipase-catalyzed decomposition of *p*-NPL as a function of the molar fraction of BMIMBF_4_ in an aqueous solution, without C_12_-MIMBF_4_ (▬) and 10 mM C_12_-MIMBF_4_ (▬). Inset: zoom of the data in the region of BMIMBF_4_ displaying lower CALB activity (region at $\chi_{BMIMBF4}$ > 0.4).

Figure 2 shows the variation in the lipase-catalyzed reaction rate at different $\chi_{BMIMBF4}$ in the presence (10 mM of C_12_-MIMBF_4_, red color in Figure 2) and absence of the surfactant (black color, in Figure 2). These results suggest that in the enzymatic reaction, i) in pure solvents (buffer phosphate, $\chi_{BMIMBF4}$ = 0.0 and BMIMBF_4_, $\chi_{BMIMBF4}$ = 1.0, respectively), the rate coefficients are negligible; ii) at $\chi_{BMIMBF4}$ = 0.1, the activity of lipase is increased with the presence of C_12_-MIMBF_4_ by 50% compared with the same reaction without the surfactant, reaching the highest value obtained (superactivity phenomena); iii) in the order 0.1 ≤ $\chi_{BMIMBF4}$ ≤ 0.4, their rate coefficients decrease close to 25% and 80%, respectively, with respect to the lipase-catalyzed rate reaction obtained at $\chi_{BMIMBF4}$ = 0.1. In the cited range of molar fractions, the rate coefficient values are overturned, being they improved without the surfactant; and iv) at $\chi_{BMIMBF4}$ > 0.4, the enzyme activity decreases systematically (see inset in Figure 2).

Figure 1 agrees with Figure 2 because the range of $\chi_{BMIMBF4}$ , where the CMC is almost constant (0.1 *≤* $\chi_{BMIMBF4}<\;0.4$ ), recorded the highest enzyme activity. So, the best activity is recorded in the presence of C_12_-MIMBF_4_ compared to pure BMIMBF_4_ or any mixture of it. The formation of micelles is being promoted by the self-aggregation of C_12_-MIMBF_4_, which is responsible for the increase in the anion–water interactions and the IL–water interface. Stamatis et al. (1999) reported that the presence of micelles provides a large increase in the interfacial area, increasing the interaction between the substrate and the enzyme active site. This significant increase in enzyme activity is known as superactivity (Spreti et al., 1999; Ventura et al., 2012; Sintra et al., 2014; Matteis et al., 2016; Calderón et al., 2019). This result is attributable to the reaction media because in water (buffer phosphate) and pure BMIMBF_4_, there are no significant responses. In mixtures of IL/water, the enzymatic activity increases, but the same mixtures in the presence of C_12_-MIMBF_4_ showed increased superactivity phenomena, suggesting a preferential solvation process (Ben-Naim, 1990; Klahn et al., 2011; Alarcón-Espósito et al., 2016; Alarcón-Espósito et al., 2017).

Furthermore, Figure 2 shows two environments close to $\chi_{BMIMBF4}$ = 0.2, being it value the border line, which is rich in water composition. For this reason, it is significant to elucidate how enzyme superactivity effects are induced by C_12_-MIMBF_4_ in IL/water mixtures. Previous studies on IL/water mixtures have demonstrated that the presence of water in the IL modifies their physical and chemical properties, for instance, viscosity, density, electrical conductivity, solvation, and solubility properties (Seddon et al., 2000; Cammarata et al., 2001; Alarcón-Espósito et al., 2015; Sánchez et al., 2018a; Sánchez et al., 2018b; Danna and Harper, 2019). Seddon et al. (2000) reported on the relevance of the HB in mixtures and their incidence in structural changes (Elaiwi et al., 1995). On the other hand, Sánchez et al. (2018c) suggested two strongly demarcated zones in BMIMBF_4_/water mixtures. One of them was rich in water, which showed strong preferential solvent effects by the aqueous phase, while the other zone predominantly shows the “anion” solvent effects displayed by the IL composition (Alarcón-Espósito et al., 2016; Sánchez et al., 2018c). The authors established a relationship between the β parameter of Kamlet–Taft ( $\beta_{KT}$ ) with $\chi_{BMIMBF4}$ , where the $\beta_{KT}$ value was related to the ability of the solvent to accept the HB (Kamlet and Taft, 1976; Kamlet et al., 1977; Kamlet et al., 1983). Then, while $\chi_{BMIMBF4}$ increases until 0.2, the $\beta_{KT}$ values increase at the same time, but since $\chi_{BMIMBF4}$ > 0.2, the $\beta_{KT}$ parameter is shown to be high but constant and close to pure BMIMBF_4_. This result agrees with that obtained by Fazio et al. (2008), who reported that high quantities of water in a mixture (IL/water) can weaken the structural network of the IL by increasing water–anion and water–water interactions, with a gradual loss of cation–anion interaction in the IL and displacing the cationic moiety. For the enzymatic reaction investigated in this study, this suggests that it takes place at low compositions of BMIMBF_4_ and the presence of large concentrations of micellar aggregates. Our results suggest that the presence of this critical composition of BMIMBF_4_ has a direct influence on the enzyme and surfactant. This environment increases the catalytic rate constant ( $k_{cat}$ ), being less efficient in pure media and other mixtures characterized by a high composition of BMIMBF_4_. In our study, a significant decrease in lipase activity is observed at BMIMBF_4_ concentrations greater than $\chi_{BMIMBF4}$ > 0.4. Ventura et al. (2012) reported that enzyme inhibition is related to strong interactions of the cation with the non-polar residues of the enzyme-active site (Constantinescu et al., 2007; Bekhouche et al., 2011), and such interactions could lead to an obstruction of the active site. Fluorescent measurements related to pyrene were reported by Sánchez et al. (2018c) for all mixtures at different molar fractions, showing that at $\chi_{BMIMBF4}$ ≤ 0.2, the polarity of the mixtures diminishes dramatically with the addition of BMIMBF_4_, as the water content in the mixture decreases. The authors suggest that $\chi_{BMIMBF4}$ ≤ 0.2 is attributable to a reaction medium with high degrees of freedom and more susceptible to establish an HB.

**FIGURE 3:**
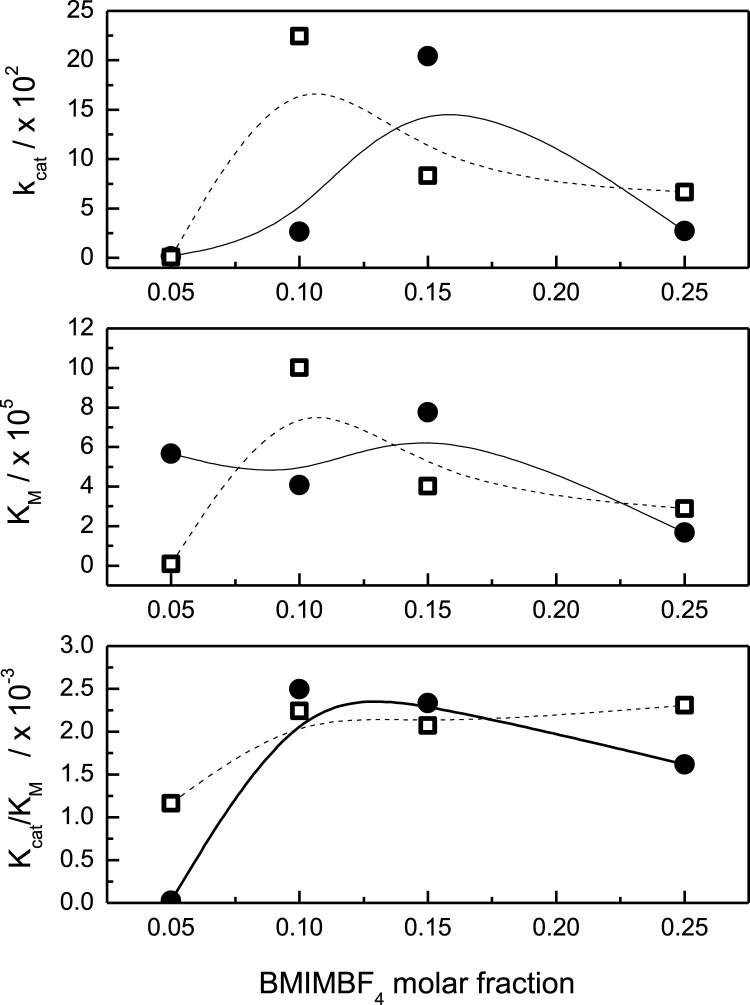
Michaelis–Menten parameters for the lipase-catalyzed solvolysis of *p*-NPL and its dependence on the molar fraction of BMIMBF_4_. Parameter calculated based on the data obtained in the presence and absence of the surfactant: no surfactant (•) and 10 mM C_12_-MIMBF_4_ (□).

Figure 3 shows the Michaelis–Menten kinetic parameters derived from the Lineweaver–Burk data analysis from the lipase activity assays (Viparelli et al., 1999; Biasutti et al., 2008; De Martino et al., 2018). The analyzed $\chi_{BMIMBF4}$ in Figure 3 corresponds to those mixtures where the lipase activity shows its higher activities (see Figure 2). In Figure 3, the plot between $k_{cat}$ vs. $\chi_{BMIMBF4}$ shows that the maximum value of $k_{cat}$ is displaced toward a lower $\chi_{BMIMBF4}$ with a maximum value at $\chi_{BMIMBF4}$ = 0.15 in the absence of the surfactant (full circles), and at 10 mM of C_12_-MIMBF_4_, the maximum value of $k_{cat}$ is located at $\chi_{BMIMBF4}$ = 0.1 (empty squares). In Figure 3, the relationship between the affinity constant, denoted by $K_{M}$ vs. $\chi_{BMIMBF4}$ , displays more variability at lower $\chi_{BMIMBF4}$ in the absence (full circles) and presence of C_12_-MIMBF_4_ (empty squares). Particularly, $K_{M}$ decreases at $\chi_{BMIMBF4}$ > 0.15. Interestingly, $K_{M}$ at $\chi_{BMIMBF4}$ = 0.1 shows a peak in the presence of C_12_-MIMBF_4_ and at $\chi_{BMIMBF4}$ = 0.15 in the absence of the surfactant. Finally, in Figure 3, the plot between $k_{cat}/K_{M}$ vs. $\chi_{BMIMBF4}$ shows the catalytic efficiency in mixtures of BMIMBF_4_ with and without C_12_-MIMBF_4_. The magnitude of $k_{cat}/K_{M}$ determined with (empty squares) and without C_12_-MIMBF_4_ (full circles) is higher in the range of $\chi_{BMIMBF4}$ between 0.1 and 0.25, with a maximum value at $\chi_{BMIMBF4}$ = 0.1 for both trends. However, at $\chi_{BMIMBF4}$ = 0.05 without C_12_-MIMBF_4_ (full circles), a decrease in catalytic efficiency is observed.

Further inspection of the Michaelis–Menten catalytic parameters shows that there is a synergistic effect between BMIMBF_4_ and the imidazolium-based surfactant. All experimental conditions considered, a complete incorporation of the substrate into the micellar moiety can be expected, with the interfacial reaction taking place at the micelle/water interface, which appears to be enhanced by the presence of BMIMBF_4_. This is particularly interesting, considering that the influence of BMIMBF_4_ in the absence of the surfactant is regarded to have a moderate/high impact on the enzyme activity. Given that, in the presence of C_12_-MIMBF_4_, the studied lipase-catalyzed reaction requires the interaction of the enzyme with the micellar surface to have access to the substrate, there are at least three possible effects responsible for the observed phenomenon:

i) High substrate concentration and its incorporation into the micelles: The enzymatic reactions can take place in micellar environments; however, the occupation of the substrate and enzyme in the micelles is lower under the experimental condition considered. Moreover, BMIMBF_4_ can influence changes in the surfactant CMC values measured, which leads to changes in the concentration of micelles in the system (assuming a constant surfactant aggregation number), but these changes do not fully correlate with the observed catalytic behavior.
ii) Increased enzymatic activity due to enhanced micellar partition of the enzyme: Similar to i), an increase in the local concentration of the enzyme on the micellar surface might lead to increased activity. However, considering a low micellar occupancy of the substrate molecules, the impact of the increased enzyme concentration should be minimal.
iii) Enhanced lipase activity due to changes in the intrinsic nature of the micellar interface: According to the available data in the present work, particularly the determination of the counterion occupancy at the micellar interface, it can be proposed that the interaction between the enzyme and the micelles, taking place at the water/micelle interface, leads to a modification of the enzyme activity attributed to conformational changes of the enzyme. This is further enhanced by the presence of BMIMBF_4_, more specifically, by the BF_4_^−^ anion, which is largely incorporated at the water/micelle interface.

**FIGURE 4:**
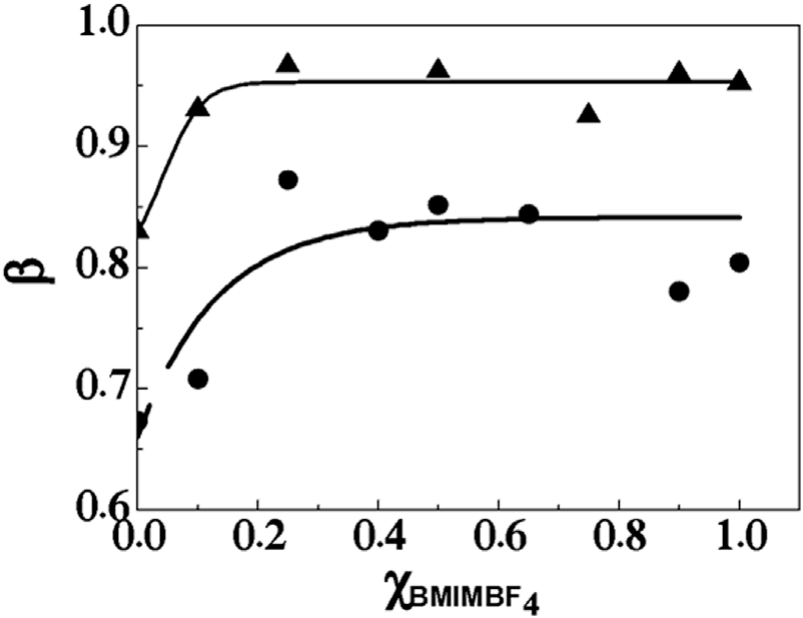
Counterion binding fraction (**β**) for micelles of C_12_-MIMBF_4_ as a function $\chi_{BMIMBF4}$ in aqueous solutions. Full circles correspond to micelles without *p*-NPL, and full triangles correspond to micelles with 0.58 mM *p*-NPL.

In order to address these possible effects, particularly those related with interfacial changes (point *iii)* in the previous paragraph) taking place in the micellar moiety, i.e., the zone delimited by the interaction between the solvent and the headgroups of C_12_-MIMBF_4_, the counterion binding to the micellar surface was determined (Khalid et al., 2017). Figure 4 shows the counterion binding fraction (β) for micelles of C_12_-MIMBF_4_ as a function of $\chi_{BMIMBF4}$ in aqueous solutions. The degree of β was calculated according to the following equation:

$$\beta =\left(1-\alpha \right),$$ (2)

where α corresponds to the ratio between the slopes of the post- and pre-CMC segments of conductivity vs. $\chi_{BMIMBF4}$ plots (please refer to Supplementary Figures S1, S2 in electronic Supplementary Material). Figure 4 shows two zones strongly demarcated. The first zone, rich in water at the range 0 ≤ $\chi_{\text{BMIMBF}4}\;0.2$ , is characterized by strong variations in β-values. This parameter suggests an increase in counterions binding to the micelle. The second zone, at $\chi_{BMIMBF4}\;>0.2$ , corresponds to a plateau, suggesting that the β-values are independent of $\chi_{BMIMBF4}$ .

Figure 4 shows differences in the micellar environment in the presence of the substrate (0.58 mM, full triangles). The β-values are at least 20% higher than without the presence of the substrate. This fact suggests that the substrate operates as a cosurfactant in the micellar environment. Then, the number of available adsorption sites for the incorporation of the surfactant counterions (BF_4_^−^) is improved. At $\chi_{BMIMBF4}\;>0.2$ , the β-value is close to unity (full triangles), suggesting that the interaction of the enzyme with the solvent/headgroup interface takes place in a surface saturated with BF_4_^−^ anions, diminishing the catalytic activity (see Figure 2) with the increase in $\chi_{BMIMBF4}$ .

**FIGURE 5:**
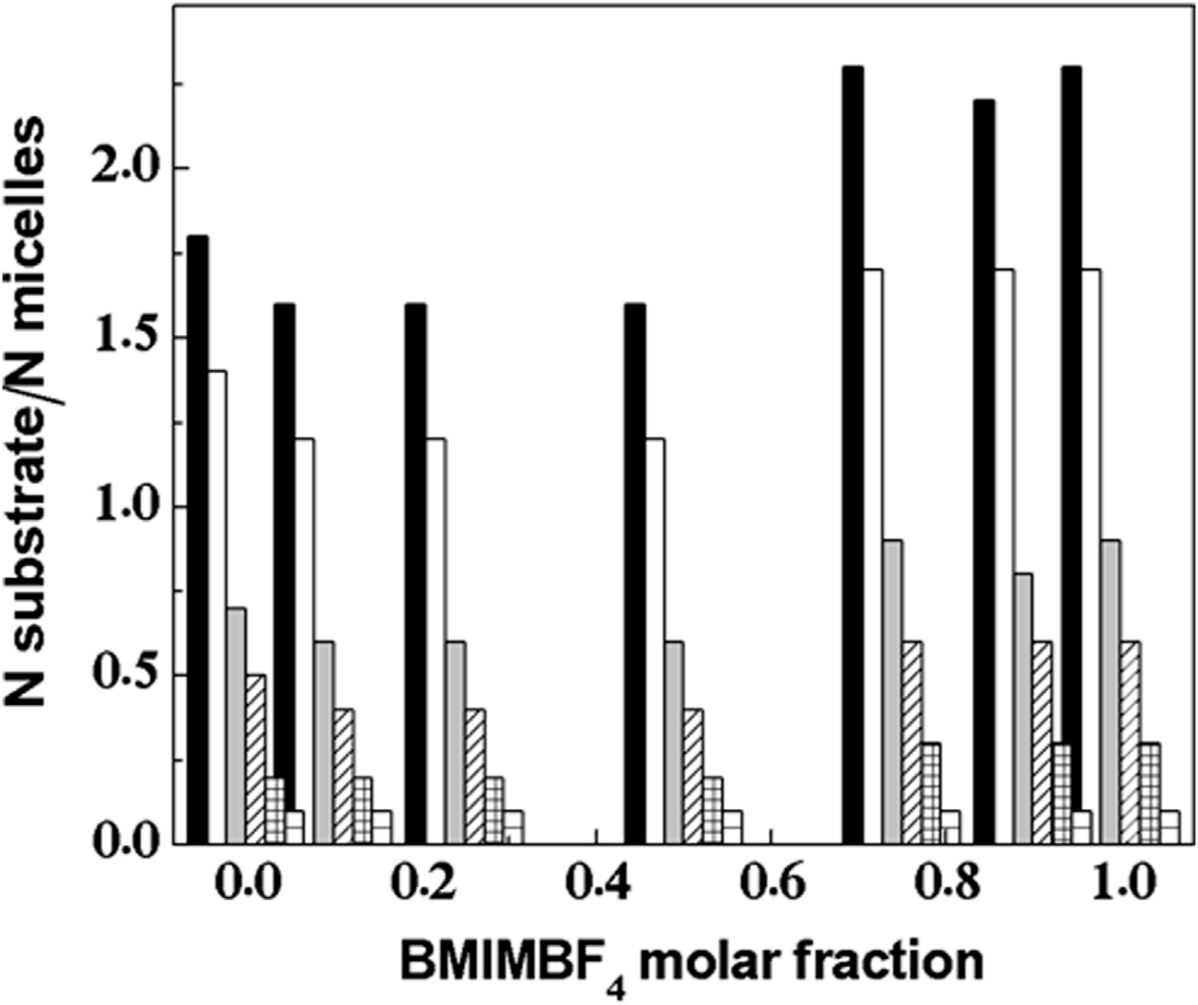
Estimated number of substrate molecules per micelle ratio as a function of BMIMBF_4_ content in the studied aqueous mixtures. Bars correspond to different substrate concentrations (left to right): 2.0 × 10^−4^ M; 1.5 × 10^−4^ M; 7.5 × 10^−5^ M; 5.0 × 10^−5^ M; 2.5 × 10^−5^ M; and 1.0 × 10^−5^ M (data are given in Supplementary Material).

Some reports have associated the interfacial phenomenon influencing the interaction between the micelle-bound substrate and lipase with the potential distribution of substrate molecules among the population of micelles present under a given experimental condition (Huang et al., 2008; Wu et al., 2008). Figure 5 shows the estimated number of substrate molecules per micelles with respect to $\chi_{BMIMBF4}$ . Equation 3 allows us to estimate the concentration of micelles calculated by means of an approximate aggregation number (N), which is determined by geometrical considerations (surfactant hydrophobic chain length and headgroup approximate size), as well as hydrodynamic radius data determined by dynamic light scattering measurements,

$$N=4\pi \frac{{\left(L_{c}+r\right)}^{2}}{a_{0}},$$ (3)

where $L_{C}$ corresponds to the surfactant chain length and r and $a_{0}$ are the radii and surface area of the surfactant headgroup, respectively.

Figure 5 shows an ideal scenario, where the substrate molecules are distributed as evenly as possible among the total number of micelles. Hence, the system is always dealing with low substrate occupancy in the micelles in the complete range of substrate concentrations considered. This indicates that the high efficiency achieved by the catalytic process takes place at the solvent/micelle interface, especially under the influence of BMIMBF_4_. On the other hand, the loss of activity observed with the increase in $\chi_{BMIMBF4}$ and its effect over the micellar solutions are not greatly affected by changes in the population of substrate molecules. This fact agrees with Figures 1, 4. Figure 1 (full triangles) shows the decreases in micelle concentration with the increase in CMC observed with the increase in $\chi_{BMIMBF4}$ . Figure 4 (full triangles) shows changes in the enzyme–micelle interaction, which might lead to loss of activity due to the inability of lipase to interact with the micellized substrate molecules, being only able to interact with substrate molecules solubilized in $\chi_{BMIMBF4}$ -rich aqueous media.

**SCHEME 2:**
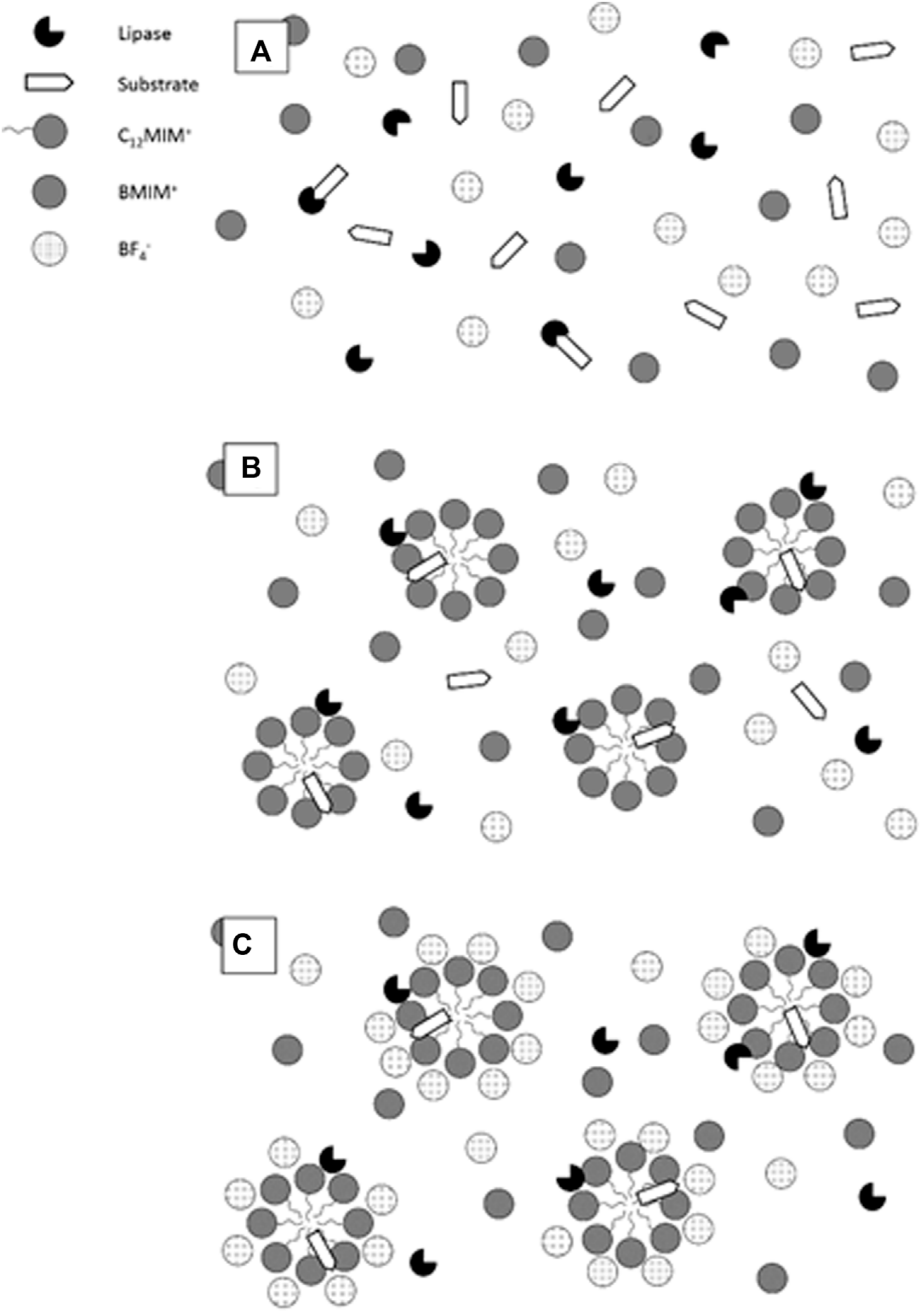
Depiction of the microenvironment for the lipase-catalyzed degradation of *p*-NPL. **(A)** Water/BMIMBF_4_ mixtures; **(B)** 10 mM C_12_-MIMBF_4_ in low-IL content water/BMIMBF_4_ mixtures; and **(C)** 10 mM C_12_-MIMBF_4_ in high-IL content water/BMIMBF_4_ mixtures.

As a plausible description of the aforementioned phenomena, Scheme 2 shows the effect of the micelles on the studied enzymatic reaction. Scheme 2 is a general picture that describes more easily the effect of the micelles on the enzymatic reaction. Scheme 2A shows the enzymatic reaction in water–BMIMBF_4_ mixtures. Schemes 2B shows the enzymatic reaction in micelles of C_12_-MIMBF_4_ in the presence of water–BMIMBF_4_ mixtures (low $\chi_{BMIMBF4}$ ). Both schemes (A and B) show the substrate molecules available for the enzyme. However, Scheme 2B shows the interfacial interaction that leads to enhanced lipase activity. BMIMBF_4_ operates directly on the changes induced in the enzyme upon its interaction with the micellar interface. Scheme 2C shows the enzymatic reaction in micelles of C_12_-MIMBF_4_ in the presence of water–BMIMBF_4_ mixtures at high $\chi_{BMIMBF4}$ . BMIMBF_4_ and C_12_-MIMBF_4_ share the imidazolium moiety and the BF_4_^−^ counterion. Then, the presence of either of these species should be responsible for the modification of the micelle-induced enzymatic activity changes. Scheme 2C shows large counterion binding to the micellar moiety, which might indicate that the changes in lipase activity derive from the large local negative charge density directly influencing the enzyme conformation. This conformational change lead to the observed activity changes, with no relevant changes in the extent and/or mechanism of interaction of the enzyme with the water/micelle interface. A pertinent alternative will be integrated to a reliable molecular dynamics study in order to support the proposed explanation to the phenomenon described for the surfactant/ionic liquid/enzyme system under study.

Finally, one of the major limitations to the specific contributions made by the surfactant molecules in micellar aggregates lies in the fact that the concentration of micelles cannot be further increased without losing a significant amount of activity, attributable mainly to amounts of the enzyme that ends up adsorbed in micelles devoid of substrate molecules, hence lowering the effective concentration of the active enzyme in the system. Additionally, large micellar concentrations lead to changes in the aggregation number and geometry of the micelles, introducing further considerations to the overall phenomenon.

## 4 Conclusion

BMIMBF_4_ can increase the catalytic rate of CALB in the hydrolysis of the *p*-NPL reaction at low $\chi_{BMIMBF4}$ , particularly in the presence of C_12_-MIMBF_4_. The significant influence of low $\chi_{BMIMBF4}$ over lipase activity suggests that specific interactions occur between BMIMBF_4_ and lipase. Fluorescence analysis reveals this zone to be rich in water with strong preferential solvent effects mediated by the aqueous phase, showing a predominant “anion” solvent effect by the IL composition. These experimental conditions suggest a complete incorporation of the substrate into the micellar moiety. Hence, the interfacial reaction takes place at the micelle/water interface, enhanced by the presence of BMIMBF_4_, attributable to conformational changes in the enzyme, and the possibility to incorporate the BF_4_^−^ anion at the water/micelle interface, thus influencing directly the interfacial catalytic performance of the enzyme.

## Data Availability

The original contributions presented in the study are included in the article/Supplementary Material; further inquiries can be directed to the corresponding author.
