# Supplementary material for: Exploring the behavior of Candida antarctica lipase B in aqueous mixtures of an imidazolium ionic liquid and its surfactant analogue
Source: Front Chem. 2024 Jan 10;11:1289398. doi: 10.3389/fchem.2023.1289398 (PMC10806215; doi:10.3389/fchem.2023.1289398)
Supplement: Supplementary file 1 [file DataSheet1.PDF]

Supplementary Material

Exploring the Behavior of *Candida antarctica* lipase B in aqueous mixtures of an imidazolium ionic liquid and its surfactant analogue.

Paola R. Campodónico[1]*, Cristian Calderón[2], Jackson Alcazar[1], Belén Olivares[1], Limberg Jaldin[1]and Cristian Suárez[1].

[1] Centro de Química Médica, Instituto de Ciencias e Innovación en Medicina, Facultad de Medicina, Clínica Alemana Universidad del Desarrollo, Santiago 7710162, Santiago, Chile.

[2] Facultad de Química y Biología, Universidad de Santiago de Chile, USACH, Santiago, Chile.

*** Correspondence:**
Corresponding Author

E-mail address: pcampodonico@udd.cl (P. R. Campodónico)

Centro de Química Médica, Instituto de Ciencias e Innovación en Medicina, Facultad de Medicina, Clínica Alemana Universidad del Desarrollo, Santiago, Chile.

Table of contents

Item	Page
**Figure S1.** Specific conductivity vs surfactant concentration plots for the determination of [$C_{12}MIMBF_4$] critical micelle concentration (CMC) in the absence of lipase synthetic substrate in water and water/$BMIMBF_4$ mixtures. From top to bottom, plots are arranged in order of increasing $BMIMBF_4$ molar fraction ($\chi_{BMIMBF4}$).	**3**
**Figure S2.** Specific conductivity vs surfactant concentration plots for the determination of [$C_{12}MIMBF_4$] critical micelle concentration (CMC) in the presence of lipase synthetic substrate in water and water/$BMIMBF_4$ mixtures. From top to bottom, plots are arranged in order of increasing $BMIMBF_4$ molar fraction ($\chi_{BMIMBF4}$).	**4**
**Table S1.** Critical micelle concentration (CMC) values determined from the inflection point in specific conductivity vs surfactant concentration plots.	**5**
**Table S2.** Slope values from initial reaction rate plots for the release of p-nitrophenol release for the lipase catalyzed hydrolysis of p-NPL in water and water/$BMIMBF_4$ mixtures, in the absence and in the presence of 10 mM $C_{12}MIMBF_4$.	**5**
**Table S3.** Michaelis-Menten parameters determined for the CALB/ p-nitrophenyl laureate system.	**6**
**Table S4.** Counterion binding fraction (β) values for $C_{12}MIMBF_4$ micelles in water and water/$BMIMBF_4$ mixtures estimated in the presence and absence of p-NPL.	**6**
**Table S5.** Calculated number of substrate (p-NPL) molecules per $C_{12}MIMBF_4$ micelle (N_{subs}/N_{mic}) water and water/$BMIMBF_4$ mixtures.	**6**
**Table S6.** Molar fractions of each misture.	**7**

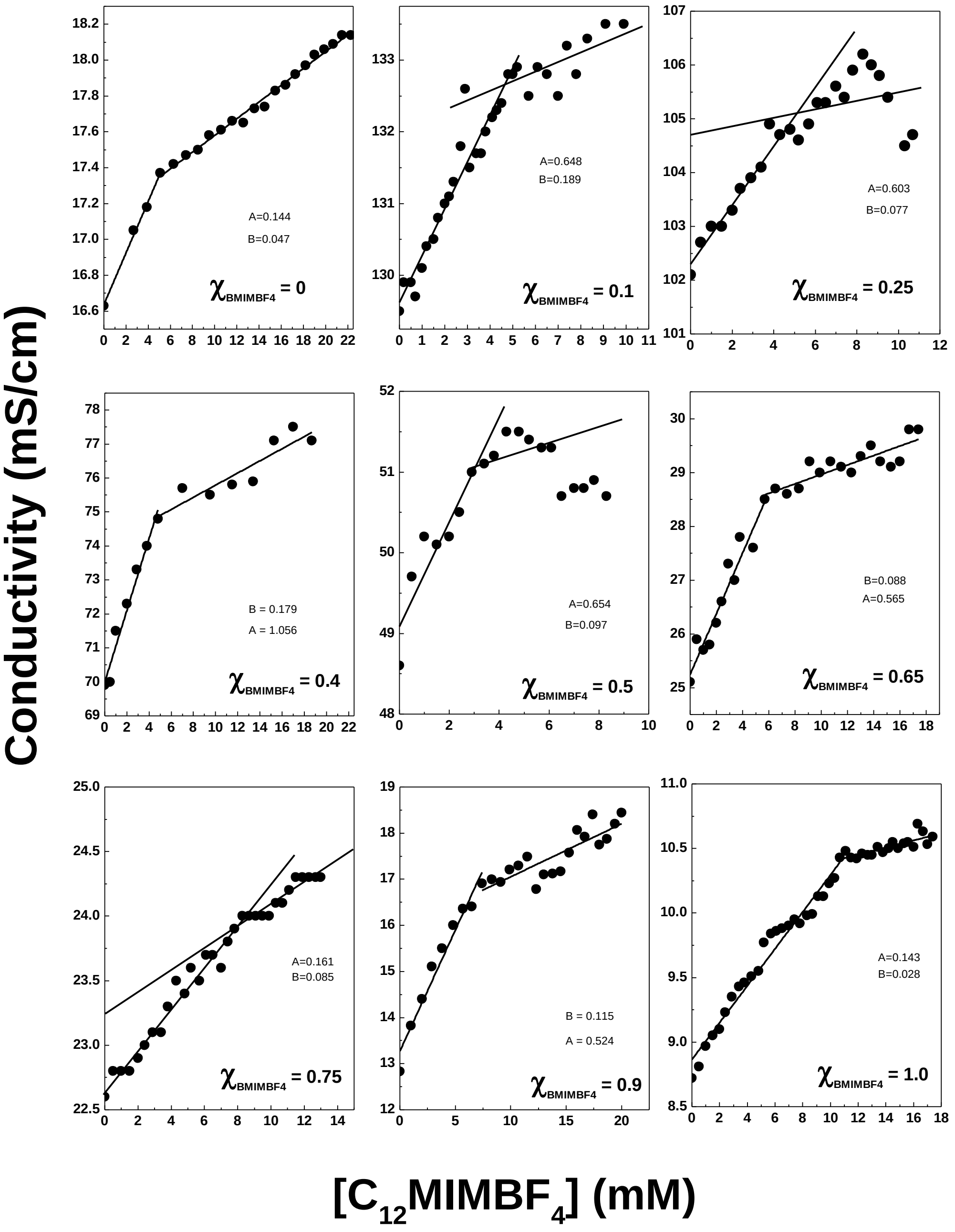

Figure S1. Specific conductivity vs surfactant concentration plots for the determination of [$C_{12}MIMBF_4$] critical micelle concentration (CMC) in the absence of lipase synthetic substrate in water and water/$BMIMBF_4$ mixtures. From top to bottom, plots are arranged in order of increasing $BMIMBF_4$ molar fraction ($\chi_{BMIMBF4}$).

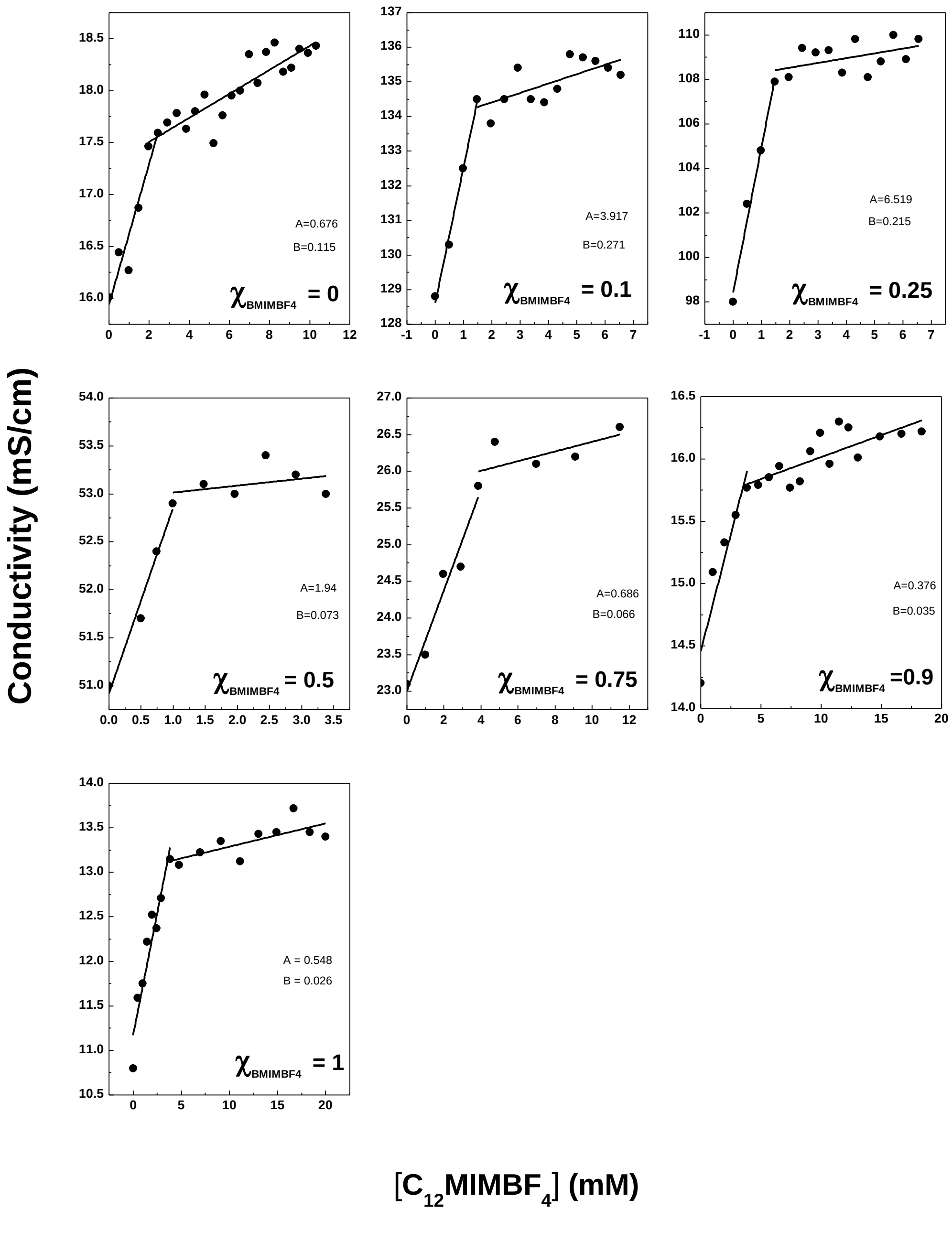

Figure S2. Specific conductivity vs surfactant concentration plots for the determination of [$C_{12}MIMBF_4$] critical micelle concentration (CMC) in the presence of lipase synthetic substrate in water and water/$BMIMBF_4$ mixtures. From top to bottom, plots are arranged in order of increasing $BMIMBF_4$ molar fraction ($\chi_{BMIMBF4}$).

Table S1. Critical micelle concentration (CMC) values determined from the inflection point in specific conductivity vs surfactant concentration plots.

$BMIMBF_4$ molar fraction (χ)	CMC	
	Substrate	No substrate
0	5.12	2.37
0.10	4.59	1.45
0.25	5.06	1.50
0.40	4.85	-
0.50	3.17	0.99
0.65	5.69	-
0.75	7.85	3.86
0.90	7.45	3.73
1	10.78	3.87

Table S2. Slope values from initial reaction rate plots for the release of p-nitrophenol release for the lipase catalyzed hydrolysis of p-NPL in water and water/$BMIMBF_4$ mixtures, in the absence and in the presence of 10 mM $C_{12}MIMBF_4$.

	[Surfactant] (mM)	
$BMIMBF_4$ molar fraction (χ)	0	10
0	1.04E-04	--
0.1	0.007	0.014
0.25	0.0055	0.003
0.50	6.80E-04	3.10E-04
0.75	1.10E-04	8.60E-05
0.90	2.48E-04	8.76E-04
1	7.98E-05	7.40E-05

Table S3. Michaelis-Menten parameters determined for the CALB/ p-nitrophenyl laureate system

$BMIMBF_4$ molar fraction (χ)	k_{cat} (s^{-1})	K_M (M^{-1})	k_{cat}/K_M (M^{-1} s^{-1})
10 mM $C_{12}MIMBF_4$			
0.05	0.0010	8.69 x 10^{-7}	1161.4
0.10	0.2243	1.00 x 10^{-4}	2242.1
0.15	0.0833	4.03 x 10^{-5}	2070.4
0.25	0.0667	2.89 x 10^{-5}	2309.5
No surfactant			
0.05	0.0015	5.66 x 10^{-5}	25.6
0.10	0.0266	4.07 x 10^{-5}	2496.1
0.15	0.2041	7.75 x 10^{-5}	2332.0
0.25	0.0270	1.67 x 10^{-5}	1616.9

Table S4. Counterion binding fraction (β) values for $C_{12}MIMBF_4$ micelles in water and water/$BMIMBF_4$ mixtures estimated in the presence and absence of p-NPL.

	β	
$BMIMBF_4$ molar fraction (χ)	No p-NPL	0.58 mM p-NPL
0	0.67	0.83
0.1	0.71	0.93
0.25	0.87	0.97
0.40	0.83	-
0.50	0.85	0.96
0.65	0.84	-
0.75	0.47	0.93
0.9	0.78	0.96
1.0	0.80	0.95

Table S5. Calculated number of substrate (p-NPL) molecules per $C_{12}MIMBF_4$ micelle (N_{subs}/N_{mic}) water and water/$BMIMBF_4$ mixtures.

	[p-NPL] (M)					
	2.00 x10^{-4}	1.50 x 10^{-4}	7.50 x 10^{-5}	5.00 x 10^{-5}	2.50 x 10^{-5}	1.00 x 10^{-5}
**$BMIMBF_4$ molar fraction (χ)**	**N_{subs}/N_{mic}**					
0	1.8	1.4	0.7	0.5	0.2	0.1
0.1	1.6	1.2	0.6	0.4	0.2	0.1
0.25	1.6	1.2	0.6	0.4	0.2	0.1
0.5	1.6	1.2	0.6	0.4	0.2	0.1
0.75	2.3	1.7	0.9	0.6	0.3	0.1
0.9	2.2	1.7	0.8	0.6	0.3	0.1
1	2.3	1.7	0.9	0.6	0.3	0.1

Table S6. Molar fraction of the mixtures defined to respect $BMIMBF_4$.

$BMIMBF_4$ (g)	Buffer (g)	$\chi_{BMIMBF4}$
20.3803	14.6197	0.1
28.2459	6.7541	0.25
32.4163	2.5837	0.5
33.5597	1.4403	0.65
34.0941	0.9058	0.75
34.6927	0.3072	0.90

The molar fraction, is defined as the ratio between the amount of a constituent substance, n_i (expressed in unit of moles) and the total amount of all constituents in a mixture, n_{tot} (also expressed in moles). In the context of this article, the molar fraction was defined to respect to $BMIMBF_4$ ($\chi_{BMIMBF4}$). Then, all the $\chi_{BMIMBF4}$ were prepared in buffer phosphate 50 mM and pH=7.0 (water) and $BMIMBF_4$ pure.

$$\chi_{BMIMBF4} = \frac{n_{BMIMBF4}}{n_{BMIMBF4} + n_{Buffer}}$$

For instance:

$$\chi_{BMIMBF4} = \frac{0.1535}{0.1535 + 0.0171} = 0.8997 = 0.90$$
